# Supplementary material for: Affinity capture of polyribosomes followed by RNAseq (ACAPseq), a discovery platform for protein-protein interactions
Source: eLife. 2018 Oct 22;7:e40982. doi: 10.7554/eLife.40982 (PMC6197854; doi:10.7554/eLife.40982)
Supplement: Supplementary file 1. — DC, plasmids from Davide Comoletti. WW, plasmids from Woj Wojtowicz. The threshold for target interaction is: (1)>35 captured TPMs, (2) captured TPMs > 3 x the maximal number of TPMs captured with any of the other baits used in the same experimental cohort, (3) captured TPMs > 3 x the TPMs in the unselected polyribosomes, and (4) the target is a secreted or membrane protein (as designated by https://www.proteinatlas.org/ humanproteome/secretome). The following baits used the mouse Fz8 signal peptide: ASTM1, ASTN2, EPO, FGF19, FGF21, VEGF-Fc. All other baits used the endogenous signal peptide. The last six amino acids of the bait sequence is shown for those baits that are derived from a protein with an amino-terminal extracellular domain followed by a trans-membrane domain. The following baits are human sequences: EPO, FGF19, FGF21, VEGF-Fc, Fc-VEGF. All others are mouse sequences. [file elife-40982-supp1.doc]

Supplementary Table 1. Baits and captured targets.

Bait (GenBank Accession; Addgene number) Number Known, confirmed,

Plasmid bait amino acids, last six bait amino acids of targets or plausible targets

pPMS1136 AGER (NM_007425) 1-334, GSVGES 0

pPMS1267 APP (NM_001198823) 1-624, DVGSNK 4 Cntn4

pPMS1268 APLP2 (U15571) 1-637, FSLSSN 4

pHC53 ASTN1 (NM_001205204) 609-1303 1

pHC54 ASTN2 (NM_207109) 665-1352 138

pMS18 AU021092 (AU021092) 1-385 2 Igf2r, Csmd3

pXP52 CDH8 (BC057581) 1-540, LPIGLS 40

pMS10 CD40 (M83312) 1-186, QTNVIC 0

pMS12 CD93 (NM_010740) 1-572, DTDGQN 2

pPMS1350 CD200R1 (NM_021325) 1-239, GNQSLR 1

pXP2 CEACAM1 (NM_001039185) 1-423, QGGLSD 8

pXP3 CEACAM 18 (NM_028236) 1-328, PTVNRE 102

pXP5 CEACAM 19 (NM_177036) 1-147, AREVSS 45 Tenm1

pXP10 CEACAM 20 (NM_027839) 1-410, VTGLAR 2

pXP48 CLSTN3 (NM_153508) 1-849, VPSAAT 122

pXP11 (DC) CNTN1 (BC066864) 1-975, QVKISG 10 Ptprz1

pXP12 (DC) CNTN3 (NM_008779) 1-2981, IRIPRI 7 App, Ptprg

pXP13 (DC) CNTN4.2 (BC115766) 1-2114, TGDQRG 14 Igf2r, Ptprz1, Ptprg

pXP14 (DC) CNTN6 (NM_017383), 1-994, IRIPKM 24

pQX62 DKK1 (NM_010051) 1-273 0

pPMS1226 DKK3 (NM_001360257) 1-367 8

pPMS1227 DKK4 (NM_145592) 1-222 1

pPMS1127 EFNA1 (NM_010107) 1-182, HSIGYS 21 Epha3, Epha4 ,Epha5 ,

Epha6 ,Epha7, Epha8

pPMS1132 ELFN1 (NM_175522) 1-416, SPSTAT 15

pPMS1133 ELFN2 (NM_001358692) 1-395, STSTTT 91

pPMS1354 ELTD1 (BC017134) 1-190, ITEQTT 2

pPMS1283 ENG (BC029080) 1-580, PDLSGK 4

pPMS1128 EPHB1 (NM_173447) 1-538, SELREQ 31 Efnb2

pPMS854 EPO (NM_000799) 28-194 51

pAR228 ESM1 (NM_023612) 1-184, KWLNPR 2

pPMS855 FGF19 (AF110400); 23-217 4

pPMS859 FGF21 (NM_019113); 30-210 22

pPMS1228 FLRT1 (NM_201411) 1-544, LNQEQN 8 Adgrl2, Adgrl3,

Unc5a, Unc5c

pPMS1229 FLRT2 (NM_201518) 1-539, SHSMGS 11 Adgrl1, Adgrl2, Adgrl3

pPMS1230 FLRT3 (AY495670) 1-526, PYKNPN 13 Adgrl2, Adgrl3, Unc5a,

Unc5b, Unc5c, Unc5d

pMS14 GPR116 (NM_001081178) 1-561, KDVTVH 2 Adgrf5

pPMS1144 GPR124 (NM_054044) 1-348, IVVLET 13

pXP27 HEPACAM2 (NM_178899), 1-349, AQRGKS 1

pXP28 IGSF11 (NM_170599) 1-239, SPQPRS 0

pXP22 INTECTIN (NM_025929) 1-87, CNTDLC 10

pXP44 (WW) ISLR2.B (NM_001161535; Addgene 72077)

1-3378, KELPSL 1

pPMS1284 ISM1 (NM_001276489) 1-461 0

pPMS1337 JAM1 (NM_172647) 1-233, HMDAVE 4

pPMS1338 JAM2 (NM_023844) 1-233, KRMQVD 2 Jam3

pPMS1339 JAM3 (NM_023277) 1-239, DMEVYD 0

pPMS1281 LDLR (AF425607) 1-790, QPHGVR 6

pPMS1224 LPHN1 (XM_011248410) 1-417, SPPLST 21 Tenm2, Tenm3, Flrt2, Flrt3, Adgra3

pPMS1225 LPHN3 (NM_198702) 1-509, GSTTTS 7 Tenm1, Tenm2, Tenm3,

Tenm4, Flrt1, Flrt2, Flrt3

pPMS1131 LRTM2 (NM_172492) 1-306, PVSVRR 3 TnR, Tnc, TnN, Ptprz1

pXP30 LSAMP (NM_001347236), 1-314, YAKTEP 0

pXP31 LSR (BC004672) 1-206, AGPLED 6

pMS11 LY75 (NM_013825) 1-1669, PLSPDY 0

pXP1 LYPD6 (BC070462) 1-149, SPINQT 50 Celsr1, Celsr2

pPMS1375 MADCAM1 (NM_013591) 1-365, PNSSST 0

pXP32 MOG (BC080860) 1-146, ELKVED 4

pPMS1242 NECL1/CADM3 (AF195662) 1-328, SSSTYH 17

pPMS1243 NECL2/CADM1 (XM_006510497)

1-378, IGAVDH 2 Cadm2

pXP45 (WW) NEO1.D (NM_001042752; Addgene 72089)

1-1132, TSPLDS 6 Sdk2, Thsd7b

pPMS1231 NLGN1(-) (NM_138666; Addgene 15260)

RDYSTE 16

pPMS1232 NLGN1(A) (NM_138666; Addgene 15227)

RDYSTE 2

pPMS1233 NLGN1(B) (NM_138666; Addgene 15261)

RDYSTE 8

pPMS1234 NLGN1(AB) (NM_138666; Addgene 15262)

RDYSTE 8

pPMS1235 NLGN2(-) (NM_001364137; Addgene 15246)

RDYSSE 11

pPMS1236 NLGN2(A) (NM_001364137; Addgene 15259)

RDYSSE 19

pAR229 NRN1 (NM_153529) 1-115, LCGSSN 1

pAR230 NRNL1 (NM_175024) 1-138, RATAPA 0

pXP42 (WW) NRP2.3 (Addgene 72100) 1-4158, LDPILI 1

pAR219 OCM (NM_033039), 1-109 2

pPMS1135 OPTC (AY077682) 1-231, LNRLQS 2

pXP53 PCDH9 (NM_001081377) 1-814, NEDYLT 6 Pcdh9

pPMS865 PDGFB (CR456538) 82-191 6

pXP41 (WW) PLXND1 (NM_026376) 1-1270, LGGSET 9

pPMS1265 PCSK9 (NM_153565) 1-695 57

pMS13 PTPRG (NM_008981) 1-733, SPGRME 3 Cntn3, Cntn4, Cntn6

pPMS1240 PVRL2 (BC009088) 1-346, PQASRD 2

pXP46 (WW) SDK2 (NM_172800; Addgene 72135)

1-1928, SPFYEE 2

pPMS1137 SEMA3A (D85028) 1-772 5

pXP39 (WW) SEMA4B (AK145570; Addgene 72155)

1-700, GADKSY 0

pPMS1244 SOST (NM_024449) 1-212 1

pPMS1273 SOSTDC1 (NM_025312) 1-207 1

pMS16 SPARC (BC019527) 1-302, NKDLVI 4 Lrp1

pPMS1351 TIGIT (NM_001146325) 1-138, AQFQTA 1

pXP40 (WW) UNC5A.A (NM_153131.4; Addgene 72176)

1-354, CLHTSS 26 Col4a1, Col4a2, Flrt3

pPMS867 VEGF-Fc (AY047581) 27-192 8 Flt1, Kdr

pPMS1356 Fc-VEGF (AY047581) 27-192 3 Flt1, Kdr, Nrp1

pPMS1342 VSIR (NM_028732) 1-189, DSDSIT 0

pPMS1332 VSIG1 (BC125278) 1-234, TSSHPE 0

pPMS1336 VSIG8 (BC064106) 1-149, DSQRVG 34

pPMS1349 VSIG10 (NM_001033311) 1-427,VKEPLN 0

pPMS1335 VSTM4 (NM_178791) 1-175, WAFFED 0

pMS17 WFDC1 (NM_023395) 1-211, QQRHFP 4
