# Supplementary material for: Affinity capture of polyribosomes followed by RNAseq (ACAPseq), a discovery platform for protein-protein interactions
Source: eLife. 2018 Oct 22;7:e40982. doi: 10.7554/eLife.40982 (PMC6197854; doi:10.7554/eLife.40982)
Supplement: Supplementary file 2. [file elife-40982-supp2.doc]

Supplementary Table 2. ACAPseq with six baits: abundance and fold enrichment of targets mRNAs

Bait Target Fold enrichment Target mRNA abundance in mouse brain polyribosomes

CNTN3 App 420-fold 0.080%

LPHN1 and Flrt1 2.5-fold 0.0021%

LPHN3 Flrt2 50-fold 0.012%

Flrt3 100-fold 0.0037%

LRTM2 Tnc 215-fold 0.0045%

Tnn 40-fold 0.00002%

Tnr 25-fold 0.0047%

LYPD6 Celsr1 250-fold 0.0011%

Celsr2 475-fold 0.012%

Celsr3 0.9-fold 0.0045%

PCDH9 Pcdh9 300-fold 0.019%

VEGF Flt1 65-fold 0.0004%

Kdr 8-fold 0.0014%

Nrp1 70-fold 0.0013%
