# Supplementary material for: Affinity capture of polyribosomes followed by RNAseq (ACAPseq), a discovery platform for protein-protein interactions
Source: eLife. 2018 Oct 22;7:e40982. doi: 10.7554/eLife.40982 (PMC6197854; doi:10.7554/eLife.40982)
Supplement: Supplementary file 3. — The PCDH9 binding data refer to Figure 9. [file elife-40982-supp3.doc]

Supplementary Table 3. Binding properties of PCDH9 mutants.

PCDH9 multi-domain deletions

pXP Domains deleted Level of homophilic binding (magnetic bead aggregation assay)

53 full ECD +++

168 cadherin domain 7 ++

167 cadherin domains 6,7 ++

156 cadherin domains 5-7 ++

155 cadherin domains 4-7 -

154 cadherin domains 3-7 -

PCDH9 single-domain deletions

pXP Domains deleted Level of homophilic binding (magnetic bead aggregation assay)

53 Full ECD +++

182 cadherin domain 1 -

151 cadherin domain 2 ++

152 cadherin domain 3 ++

153 cadherin domain 4 -

183 cadherin domain 5 ++

184 cadherin domain 6 ++

185 cadherin domain 7 ++
